# Comparison of obesity-related indicators for identifying metabolic syndrome among normal-weight adults in rural Xinjiang, China

**DOI:** 10.1186/s12889-022-14122-8

**Published:** 2022-09-12

**Authors:** Le-yao Jian, Shu-xia Guo, Ru-lin Ma, Jia He, Dong-sheng Rui, Yu-song Ding, Yu Li, Xue-ying Sun, Yi-dan Mao, Xin He, Sheng-yu Liao, Heng Guo

**Affiliations:** 1 Department of Public Health, Shihezi University School of Medicine, North 2th Road, Shihezi, Xinjiang, 832003 China; 2 NHC Key Laboratory of Prevention and Treatment of Central Asia High Incidence Diseases, First Affiliated Hospital, School of Medicine, Shihezi University, Shihezi, Xinjiang, 832000 China

**Keywords:** Obesity-related indicators, Metabolic syndrome, Normal-weight, Screening

## Abstract

**Background:**

This study aimed to compare the ability of certain obesity-related indicators to identify metabolic syndrome (MetS) among normal-weight adults in rural Xinjiang.

**Methods:**

A total of 4315 subjects were recruited in rural Xinjiang. The questionnaire, biochemical and anthropometric data were collected from them. Binary logistic regression was used to analyze the association between the z-score of each index and MetS. The area under the receiver-operating characteristic (ROC) curves were used to compare the diagnostic ability of each index. According to the cut-off value of each index, nomogram models were established and their diagnostic ability were evaluated.

**Results:**

After adjusting for confounding factors, each indicator in different genders was correlated with MetS. Triglyceride-glucose index (TyG index) showed the strongest association with MetS in both males (OR = 3.749, 95%CI: 3.173–4.429) and females (OR = 3.521,95%CI: 2.990–4.148). Lipid accumulation product (LAP) showed the strongest diagnostic ability in both males (AUC = 0.831, 95%CI: 0.806–0.856) and females (AUC = 0.842, 95%CI: 0.820–0.864), and its optimal cut-off values were 39.700 and 35.065, respectively. The identification ability of the TyG index in different genders (males AUC: 0.817, females AUC: 0.817) was slightly weaker than LAP. Waist-to-height ratio (WHtR) had the similar AUC (males: 0.717, females: 0.747) to conicity index (CI) (males: 0.734, females: 0.749), whereas the identification ability of a body shape index (ABSI) (males AUC: 0.700, females AUC: 0.717) was relatively weak. Compared with the diagnostic ability of a single indicator, the AUC of the male nomogram model was 0.876 (95%CI: 0.856–0.895) and the AUC of the female model was 0.877 (95%CI: 0.856–0.896). The identification ability had been significantly improved.

**Conclusion:**

LAP and TyG index are effective indicators for identifying MetS among normal-weight adults in rural Xinjiang. Nomogram models including age, CI, LAP, and TyG index can significantly improve diagnostic ability.

## Introduction

Metabolic syndrome (MetS) is a cluster of cardiometabolic risk, including central obesity, elevated blood pressure, abnormal glucose tolerance, and abnormal lipid levels. Previous research has shown that the prevalence of MetS was 24.5% among people over 15 years old in China, and the prevalence increases with age [1]. A meta-analysis of 87 studies indicates that MetS could lead to a 2-fold increase in cardiovascular disease (CVD) risk and a 1.5-fold increase in all-cause mortality [2]. Therefore, early identification of individuals with MetS is important for preventing CVD and improving the health level of the population.

Research has shown that Asian populations are more prone to visceral fat accumulation (VAT) [3], which is a basic pathogenic component of MetS [4]. CT and MRI are the gold standards for detecting visceral fat distribution [5]. However, they are not suitable for large-scale population screening due to their high price and complicated steps. Currently, body mass index (BMI) and waist circumference (WC) are the most commonly used predictors, but BMI does not reflect body shape and fat distribution, whereas individuals with similar BMI may exhibit different levels of fitness [6]. In addition, cardiometabolic risks in people with normal BMI are often overlooked, with more than one-third of normal-weight Chinese adults suffering from mild to moderate cardiometabolic diseases [7]. WC is more accurate than BMI in assessing cardiometabolic risks [8]. Studies have shown that waist-to-height ratio (WHtR) is superior to BMI and WC in predicting cardiometabolic risk factors [9, 10]. Although WC and WHtR can reflect body shape to a certain extent, they cannot distinguish the distribution of fat and muscle tissue. Accordingly, it is necessary to find more suitable indicators to better evaluate central obesity and identify MetS.

At present, some emerging anthropometric indicators have been performed well to reflect cardiometabolic risks such as a body shape index (ABSI) and conicity index (CI). Wang et.al [11]. found that ABSI was the best anthropometric index to assess CHD risk in Chinese adult males. CI performed well in assessing 10-year cardiovascular events in the Iranian population [12]. Lipid accumulation product (LAP) is calculated by triglyceride and WC, which has the highest diagnostic accuracy of MetS in middle-aged and elderly people in Korea [13]. The triglyceride-glucose index (TyG index) is an emerging index that uses fasting blood glucose and fasting triglyceride to evaluate insulin resistance, meanwhile, it has a good performance in predicting CVD [14, 15]. A study including 109,551 Chinese people showed that the prevalence of MetS was higher in less educated populations and less economically developed areas [16]. Ying’s study [17] and Ma’s study [18] yielded similar results. Xinjiang is located in the northwestern of China, and the rural areas of Xinjiang have a lower economic level than southeastern regions. Therefore, compared with developed regions, the use of simple and efficient indicators to screen for MetS and cardiometabolic risk in rural areas has more important public health significance. In addition, there is no relevant report on the identification ability of the above indicators on the MetS of normal-weight adults in rural areas of Xinjiang.

Thus, this study aimed to describe the prevalence of MetS among normal-weight adults in rural Xinjiang, compare the identification ability of each indicator in different genders, and calculate the cut-off values. Finally, we build up nomogram models for different genders based on the cut-off values.

## Materials and methods

### Study population and data collection

This research was carried out in the 51st Regiment, 3rd Division, Xinjiang Production and Construction Crops from June to August 2016. Cluster random sampling was used to select the harmonious community, beautiful community, 6th company, 8th company, and hospital medical examination center of the 51st regiment as the research site. We enrolled 12,813 participants. Inclusion criteria were: 1) Residents (or living in the local area for more than 6 months). 2) Age ≥ 18 years. 3) Normal weight (BMI 18.5–24.0). Exclusion criteria were: 1) Those who were unable to complete the questionnaire, physical examination, blood sample collection, and blood pressure measurement. 2) Pregnant woman. 3) People with serious illnesses. Finally, according to the inclusion and exclusion criteria, this study included 4315 participants.

The investigation was approved by the ethical review committee of the First Affiliated Hospital of Medical College in Shihezi University (no. shz2010ll01). All subjects signed informed consent before taking part in this study. All experimental protocols for involving human data were in accordance to the Declaration of Helsinki.

### Data collection

Each participant was interviewed face to face. The standard questionnaire included age, education, occupation, marital status, smoking and drinking habits, disease history, and family history of cardiometabolic diseases and CVD. Smoking was defined as smoking more than 100 cigarettes ever [19]. Drinking was defined as drinking alcoholic beverages at least twice a month [20].

### Anthropometric measurements

The height and weight were measured by an automatic height-weight scale. Shoes, caps, and coats were taken off during measurement, and the accuracy was 0.1 cm and 0.1 kg, respectively. WC was measured at the end of expiration using nonelastic measure tapes at the midpoint between the lowest point of the rib and the upper border of the iliac crest. BMI was calculated by dividing the subject’s weight (kg) by the square of the height (m^2^). Waist-to-height ratio (WHtR) was calculated as WC (cm)/height (cm). ABSI, CI, LAP, and TyG index were calculated by the following formulas [21–24]:

$${\displaystyle \begin{array}{l}\mathrm{ABSI}=\frac{\mathrm{WC}}{{\mathrm{BMI}}^{2/3}{\mathrm{height}}^{1/2}}\\ {}\mathrm{CI}=\frac{\mathrm{WC}}{0.109\times \sqrt{\mathrm{weight}/\mathrm{height}}}\\ {}\begin{array}{l}\mathrm{LAP}\ \left(\mathrm{males}\right)=\left[\mathrm{WC}-65\right]\times \mathrm{TG},\mathrm{LAP}\left(\mathrm{females}\right)=\left[\mathrm{WC}-58\right]\times \mathrm{TG}\\ {}\mathrm{TyG}\ \mathrm{index}=\mathrm{Ln}\left[\mathrm{fasting}\ \mathrm{TG}\times \mathrm{fasting}\ \mathrm{glucose}/2\right]\end{array}\end{array}}$$

### Clinical and biochemical tests

After the subjects sat and rested for at least 5 minutes, the systolic and diastolic blood pressure were measured with Omron electronic sphygmomanometers (model HEM-7051). The average of three measurements was taken, and the interval between each measurement was 30 seconds. After fasting for at least 10 hours the night before, whole blood was drawn the next morning, anticoagulated with heparin sodium, shaken, centrifuged at 3000 r/min for 10 min, and then placed at − 20 °C for cryopreservation. Centralized low-temperature transport to the key laboratory of Shihezi University School of Medicine − 80 °C refrigerator for low-temperature storage. All biochemical indicators were detected by automatic biochemical analyzers (Olympus AU 2700; Olympus Diagnostics, Hamburg, Germany) in the Laboratory Department of the First Affiliated Hospital of Shihezi University School of Medicine.

### Definition of MetS

The definition of MetS in this study is based on the criteria defined by the joint interim statement (JIS criteria) [25]. MetS was defined as meeting ≥3 of the following components: 1) WC ≥ 85 cm for males or ≥ 80 cm for females; 2) Fasting TG ≥ 1.7 mmol/L; 3) blood pressure ≥ 130/85 mmHg or hypertension has been diagnosed and treated; 4) fasting plasma glucose (FPG) ≥ 5.6 mmol/L; 5) fasting HDL-C < 1.00 mmol/L for males or < 1.30 mmol/L for females.

### Statistical analysis

Continuous variables were described by mean ± standard deviation (SD), and categorical variables were described by frequency and percentage. Two independent samples t-tests and person χ^2^ tests were used to compare continuous variables and categorical variables in different groups, respectively. Z-scores were used for obesity-related indicators. Binary logistic regression was used to analyze the association between MetS and various indicators, adjusting for age, education, occupation, marital status, smoking, and drinking habits. The area under the receiver-operating characteristic (ROC) curves were used to evaluate the diagnostic ability of each index. The sensitivity, specificity, Youden’s index, and the cut-off value of each index were calculated. According to the determined optimal cut-off value of indicators, binary variables were constructed. Univariate logistic regression was used to select the statistically significant variables in age, occupation, marital status, smoking and drinking habits, and constructed variables. The variables with statistical significance were included in the multivariate logistic regression models, and the backward LR method was used (the inclusion criterion was *P* < 0.05, and the exclusion criterion was *P* > 0.1) to construct nomogram models. Nomograms were constructed to measure the nomogram models, and calibration curves were plotted to assess the calibration of the nomograms. All statistical analyses were stratified by sex and performed using SPSS 26.0 (SPSS Inc., Chicago, IL, USA) and R statistical software (version 4.1.2, http://www.r-project.org/). *P* < 0.05 was considered to be statistically significant.

## Results

### Basic characteristics of the study population

A total of 4315 normal-weight subjects participated in this study (2174 for males and 2141 for females). The prevalence of MetS was 16.0% (14.2% for males and 17.8% for females). For different genders, the MetS group showed significantly higher values for age, weight, WC, BMI, WHtR, ABSI, CI, LAP, TyG index, blood pressure, and serum lipid indexes (expect HDL-C) than those in the non-MetS group (All *P* values < 0.05) (Table 1).

**Table 1:** Basic Characteristics of the study participants according to gender and MetS

Characteristics	Male (*n* = 2174)			Female (*n* = 2141)		
	Non-MetS	MetS	*P-*value	Non-MetS	MetS	*P-*value
*N* (%)	1865 (85.8%)	309 (14.2%)		1759 (82.2%)	382 (17.8%)	
Age (years)	31.44 ± 13.28	39.51 ± 16.29	< 0.001	29.72 ± 11.09	42.17 ± 15.75	< 0.001
Height (cm)	170.20 ± 7.01	171.10 ± 7.46	0.038	160.02 ± 6.96	159.34 ± 8.01	0.123
Weight (kg)	62.94 ± 6.00	65.14 ± 6.29	< 0.001	55.21 ± 5.86	56.39 ± 6.33	< 0.001
WC (cm)	83.39 ± 10.67	91.95 ± 11.38	< 0.001	79.32 ± 11.37	87.51 ± 9.03	< 0.001
BMI (kg/m^2^)	21.71 ± 1.43	22.23 ± 1.32	< 0.001	21.53 ± 1.50	22.16 ± 1.28	< 0.001
WHtR	0.49 ± 0.06	0.54 ± 0.69	< 0.001	0.50 ± 0.07	0.55 ± 0.06	< 0.001
ABSI	0.0822 ± 0.0100	0.0891 ± 0.0120	< 0.001	0.0812 ± 0.0114	0.0881 ± 0.0010	< 0.001
CI	1.02 ± 0.13	1.12 ± 0.14	< 0.001	0.99 ± 0.14	1.09 ± 0.11	< 0.001
LAP	23.09 ± 20.99	61.50 ± 47.22	< 0.001	22.99 ± 19.82	61.08 ± 44.12	< 0.001
TyG index	8.25 ± 0.61	9.00 ± 0.75	< 0.001	8.07 ± 0.60	8.86 ± 0.68	< 0.001
SBP (mmHg)	122.71 ± 15.41	136.29 ± 15.77	< 0.001	117.56 ± 13.94	134.02 ± 19.68	< 0.001
DBP (mmHg)	70.51 ± 10.71	77.23 ± 11.68	< 0.001	71.01 ± 10.07	76.73 ± 12.28	< 0.001
FPG (mmol/L)	4.60 ± 0.97	5.53 ± 1.56	< 0.001	4.45 ± 0.95	5.33 ± 1.91	< 0.001
TC (mmol/L)	4.27 ± 1.21	4.76 ± 1.23	< 0.001	4.17 ± 0.99	4.81 ± 1.28	< 0.001
TG (mmol/L)	1.24 ± 0.81	2.31 ± 1.43	< 0.001	1.08 ± 0.70	2.03 ± 1.19	< 0.001
LDL-C (mmol/L)	2.43 ± 0.81	2.57 ± 0.76	0.004	2.28 ± 0.82	2.65 ± 0.90	< 0.001
HDL-C (mmol/L)	1.60 ± 0.55	1.50 ± 0.63	0.010	1.69 ± 0.55	1.39 ± 0.53	< 0.001
High BP level, (n/%)	589 (31.6%)	261 (84.5%)	< 0.001	332 (18.9%)	258 (67.5%)	< 0.001
Abdominal obesity, (n/%)	754 (40.4%)	272 (88.0%)	< 0.001	816 (46.4%)	354 (92.7%)	< 0.001
Dysglycemia, (n/%)	185 (9.9%)	165 (53.3%)	< 0.001	127 (7.2%)	143 (37.4%)	< 0.001
High TG level, (n/%)	312 (16.7%)	220 (71.2%)	< 0.001	198 (11.3%)	238 (62.3%)	< 0.001
Low HDL-C level, (n/%)	129 (6.9%)	69 (22.3%)	< 0.001	422 (24.0%)	254 (66.5%)	< 0.001

### Binary logistic regression of obesity-related indicators and MetS

**Fig. 1:**
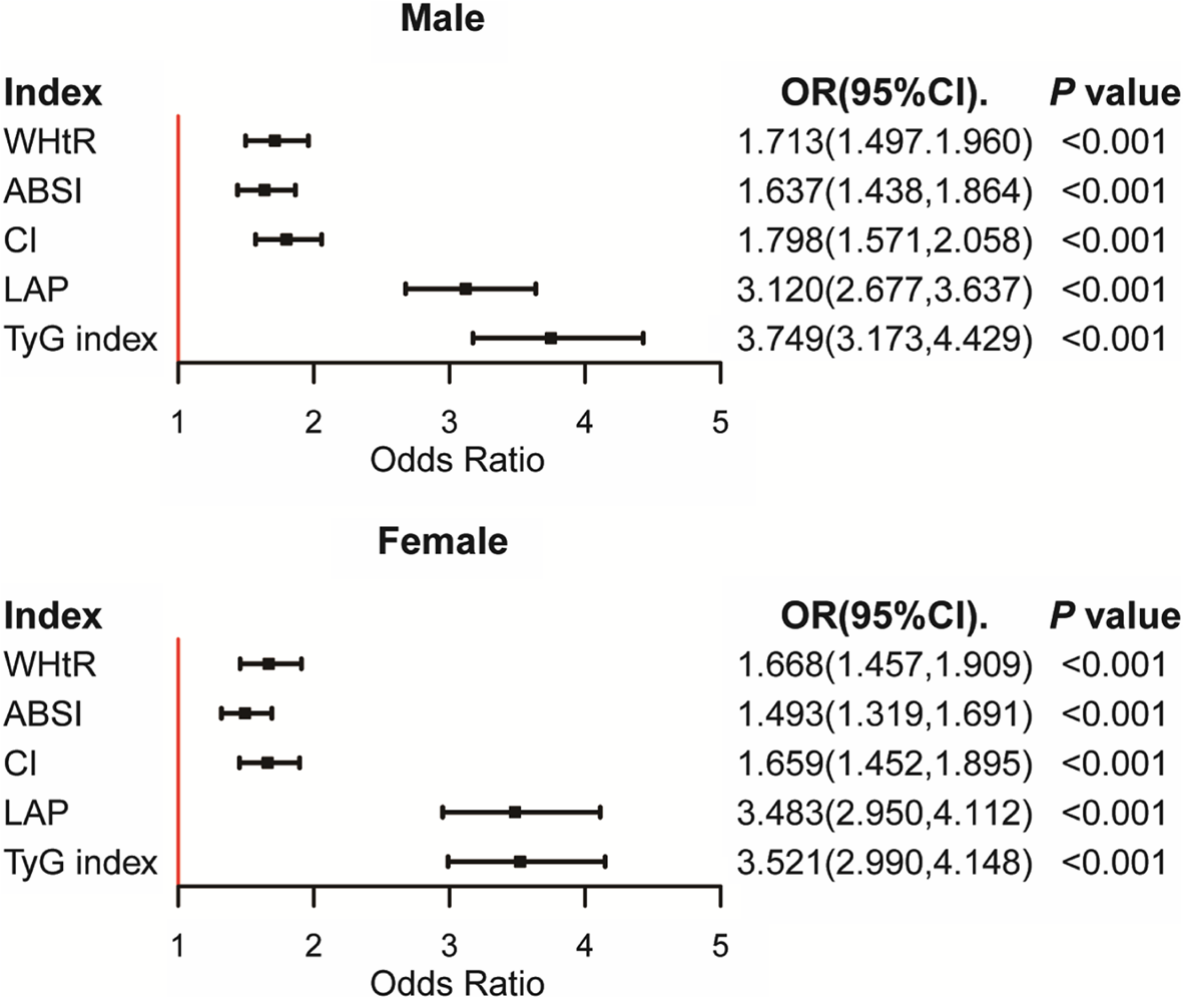
Adjusted OR and 95% CI of MetS according to the levels of each index for Male and Female. **A** Male. **B** Female. Model: Adjusted age, education, occupation, marital status, smoking, and drinking habits. **WHtR**: waist-to-height ratio, **ABSI**: a body shape index, **CI**: conicity index, **LAP**: lipid accumulation product, **TyG index**: triglyceride-glucose index

The odds ratio (OR) and 95% confidence interval (CI) were analyzed by obesity-related Z-scores after controlling for age, education, occupation, marital status, smoking, and drinking habits. All indicators were independently correlated with MetS. The TyG index had the strongest association with MetS in both males (OR = 3.749, 95%CI:3.173–4.429) and females (OR = 3.521, 95%CI: 2.990–4.148). In addition, ABSI had the weakest association with MetS (males: OR = 1.637, 95%CI: 1.438–1.864; females: OR = 1.493, 95%CI: 1.319–1.691) (Fig. 1).

### The diagnostic ability of obesity-related indicators for MetS

**Fig. 2:**
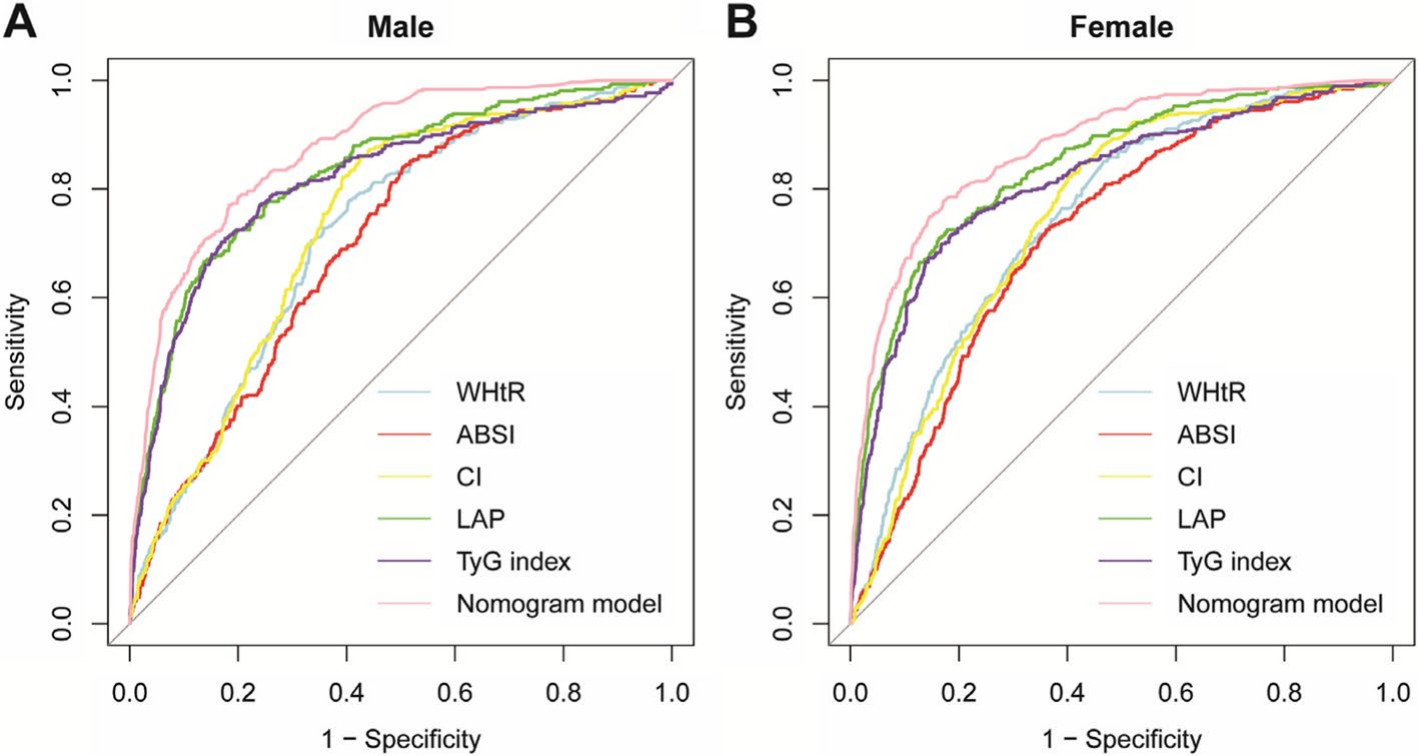
ROC curves for screening MetS for different genders. **A** Male. **B** Female. **WHtR**: waist-to-height ratio, **ABSI**: a body shape index, **CI**: conicity index, **LAP**: lipid accumulation product, **TyG index**: triglyceride-glucose index. **Nomogram model**: including age, CI, LAP, and TyG index

In males, LAP had the largest area under the curve (AUC) value of 0.831 (95%CI:0.806–0.856). The optimal cut-off value of LAP in males was 39.700 based on Youden’s index of 0.530 (sensitivity = 0.725, specificity = 0.863). But TyG index had the maximum Youden’s index of 0.533. CI had the best sensitivity at 0.858, and LAP had the best specificity. Similarly, LAP had the largest AUC (0.842, 95%CI: 0.820–0.864) in females. The optimal cut-off value for LAP in females was 35.065 based on the maximum Youden’s index of 0.546 (sensitivity = 0.725, specificity = 0.821). WHtR had the best sensitivity at 0.851, and the TyG index had the largest specificity at 0.832. There were statistically significant differences between WHtR and other indicators except for ABSI in males and CI in females. LAP and other indicators (except the TyG index) were statistically significant in both males and females. The diagnostic ability of the LAP and TyG index is better than of all other indicators (Fig. 2, Table 2).

**Table 2:** ROC analysis of each obesity-related index and Nomogram model by gender

Gender	Variables	Cut-off	Sensitivity	Specificity	Youden’s index	AUC (95%CI)	*P-*value
Male	WHtR	0.497	0.780	0.594	0.374	0.717*(0.689–0.746)	< 0.001
	ABSI	0.081	0.841	0.496	0.337	0.700*(0.671–0.729)	< 0.001
	CI	1.030	0.858	0.575	0.433	0.734^*(0.706–0.761)	< 0.001
	LAP	39.700	0.667	0.863	0.530	0.831^(0.806–0.856)	< 0.001
	TyG index	8.762	0.709	0.824	0.533	0.817^(0.788–0.845)	< 0.001
	Nomogram model	−1.915	0.770	0.817	0.587	0.876^*(0.856–0.895)	< 0.001
Female	WHtR	0.497	0.851	0.534	0.385	0.747*(0.721–0.772)	< 0.001
	ABSI	0.083	0.725	0.639	0.364	0.717^*(0.691–0.743)	< 0.001
	CI	0.998	0.848	0.575	0.423	0.749*(0.724–0.773)	< 0.001
	LAP	35.065	0.725	0.821	0.546	0.842^(0.820–0.864)	< 0.001
	TyG index	8.614	0.699	0.832	0.531	0.817^*(0.791–0.842)	< 0.001
	Nomogram model	−1.579	0.777	0.829	0.606	0.877^*(0.857–0.896)	< 0.001

**Notes**: ^ indicates *P* < 0.05 for AUC vs WHtR, * indicates *P* < 0.05 for AUC vs LAP. **WHtR**: waist-to-height ratio, **ABSI**: a body shape index, **CI**: conicity index, **LAP**: lipid accumulation product, **TyG index**: triglyceride-glucose index. **Nomogram model**: including age, CI, LAP, and TyG index

**Fig. 3:**
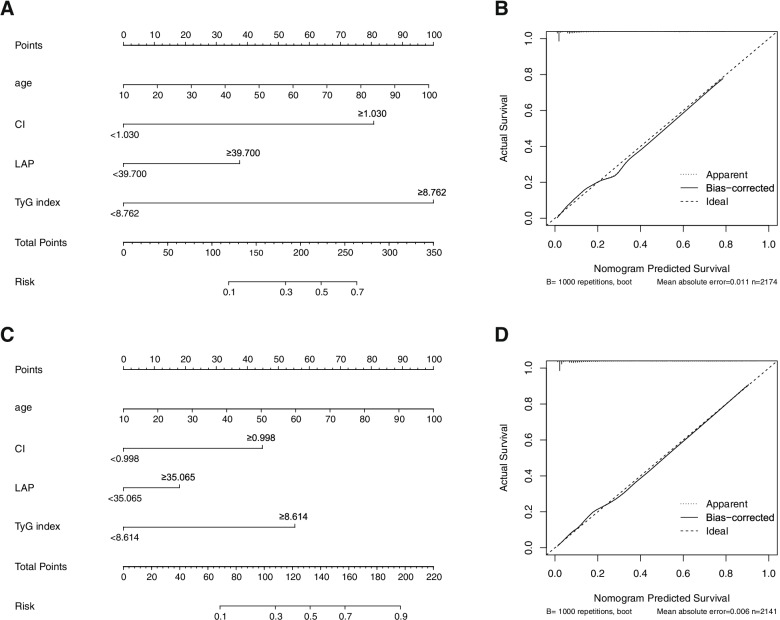
Nomogram and Calibration curves to estimate the risk of MetS for Male and Female. **A**/**B** – Male. **C**/**D** Female. **WHtR**: waist-to-height ratio, **ABSI**: a body shape index, **CI**: conicity index, **LAP**: lipid accumulation product, **TyG index**: triglyceride-glucose index. **Usage example**: If a woman was 50-year-old, 45 points can be accumulated according to (**C**). Assuming her CI ≥ 0.998, 45 points were accumulated. Similarly, assuming her LAP < 35.565 and TyG index ≥8.614, then she should accumulate 0 points and 57.5points. Then her total score was 147.5 (45 + 45 + 57.5). Finally, the risk of MetS was about 60% after making a straight line from the total points to the risk axis

After constructing binary categorical variables according to the cut-off values of obesity-related indicators, nomogram models were developed after screening variables using univariate and multivariate logistic regression (Table 3). The nomograms, calibration curves, and ROC curves of the nomogram models were plotted (Fig. 2, Fig. 3). The male nomogram model had an AUC of 0.876 (95%CI: 0.856–0.895), and the C-index was 0.875 (95% CI: 0.856–0.895). The female nomogram model had an AUC of 0.877 (95% CI: 0.857–0.896), and the C-index was 0.877 (95% CI: 0.857–0.896). In both males and females, the diagnostic ability of the nomogram models was superior to that of all obesity-related indicators (There was a significant difference in pairwise comparisons with the AUC maximum index: LAP) (Table 2).

**Table 3:** Multivariate logistic regression screening variables

Gender	Variables	OR (95%CI)	*P-*value
Male	Age	1.023 (1.013 ~ 1.033)	< 0.001
	CI	5.476 (3.675 ~ 8.159)	< 0.001
	LAP	2.202 (1.531 ~ 3.168)	< 0.001
	TyG index	8.210 (5.764 ~ 11.693)	< 0.001
Female	Age	1.039 (1.029 ~ 1.049)	< 0.001
	Drink	1.460 (0.894 ~ 2.167)	0.060
	CI	4.936 (3.424 ~ 7.117)	< 0.001
	LAP	1.893 (1.312 ~ 2.733)	< 0.001
	TyG index	6.740 (4.727 ~ 9.610)	< 0.001

**Notes**: **WHtR**: waist-to-height ratio, **ABSI**: a body shape index, **CI**: conicity index, **LAP**: lipid accumulation product, **TyG index**: triglyceride-glucose index

## Discussion

In this study, we compared the diagnostic ability of five obesity-related indicators for MetS among normal-weight adults in rural areas of Xinjiang. LAP and TyG index had the best identification ability in both males and females. Among the remaining three anthropometric indicators, WHtR and CI had stronger identification ability than ABSI. We constructed nomogram models for different genders including age, CI, LAP, and TyG index. The diagnostic ability had been significantly improved.

Research has shown that normal-weight obesity is associated with an increased cardiovascular risk [26]. In South Africa, people with a normal BMI had a higher risk of all-cause mortality than those who were overweight and obese [27]. People often overlook their own metabolic risk because of a normal BMI. Therefore, it is necessary to carry out metabolic risk screening for the normal-weight population. VAT plays an important role in the deterioration of metabolic status [4, 28]. CT and MRI are currently recognized as the gold standards for the detection of VAT [5]. However, this study was carried out in the rural area of Xinjiang in the northwest of China. It is not realistic to conduct large-scale CT and MRI examinations in this population due to the economic level and complex examination procedures. At present, the most commonly used indicators for evaluating visceral fat are BMI and WC. But BMI predicts all-cause mortality in opposite direction under certain circumstances in a 22-year cohort study [29]. BMI cannot reflect body shape and fat distribution. Although WC can reflect body shape to a certain extent, it cannot distinguish the distribution of muscle and adipose tissue. In conclusion, BMI and WC may not be good predictors of cardiometabolic risk. As a simple anthropometric indicator, WHtR is better than BMI and WC in predicting cardiometabolic risk [9, 10, 30]. It is widely used to predict cardiometabolic disease. Wu et al. [31] suggested WHtR as an early screening method for MetS in non-overweight/obese subjects. Similar results were obtained in our study, with WHtR having a relatively good diagnostic ability for MetS. The AUC for males and females were 0.717 and 0.747, respectively.

LAP is a mathematical model developed by taking into account WC and TG. Previous studies have shown that it is a better predictor of MetS for the Chinese elderly [32] and Malaysian vegetarians [33]. In our study, regardless of gender, LAP performed well in identifying MetS, which can be defined as an “excellent” indicator (0.8 ≤ AUC < 0.9) according to the criteria of Hosmer and Lemesow. This result was consistent with the above studies. The excellent diagnostic ability may be due to the inclusion of TG in the calculation of LAP, and elevated TG levels are one of the conditions for the diagnosis of MetS. The diagnostic ability of LAP for MetS in our study was lower than the Chinese elderly [32] (males AUC = 0.897, females AUC = 0.875) and Malaysian vegetarians (AUC = 0.920) [33]. The possible reason is that the subjects included in this study were normal-weight people, whose metabolic status was relatively good. The cut-off values of LAP predicted MetS in Chinese ≥60 years old people were 26.35 for males, and 31.04 for females [32]. In this study, the cut-off values were 39.700 and 35.065in males and females, respectively. The reason for the difference is that the study population is younger than the above populations and the inclusion criteria in this study are normal-weight residents. Overall, LAP has the most accurate diagnostic ability which only needs to be derived from WC and fasting TG. It is a simple and effective indicator for predicting MetS among normal-weight individuals.

TyG index has a good performance in predicting insulin resistance [34]. TyG index outperforms in predicting MetS among Taiwanese populations [35]. And TyG index can effectively predict MetS in the Nigerian population [36]. In our study, the AUC of the TyG index was 0.817 both in males and females, and the diagnostic ability was slightly weaker than that of LAP. It also can be defined as an “excellent” index according to the criteria of Hosmer and Lemesow. The result is similar to those found in a study of middle-aged and elderly populations in Korea [13]. TyG index is calculated from fasting TG and fasting FPG, which are routine indicators that can be obtained in the free health examination of the whole people. Therefore, it is also a simple and effective indicator.

Both ABSI and CI can be calculated from height, weight, and WC. A higher ABSI indicates a higher-than-expect WC for a given height and weight, reflecting more centrally the accumulation of body volume [21]. CI is based on geometric theory, that is, with the accumulation of waste fat, the body shape changes from a “cylinder” to a double “cone” [22], which can reflect the level of central obesity. Previous studies have shown that ABSI and CI perform well in predicting CHD and cardiovascular events, respectively [11, 12]. However, not all studies yielded the same results. A systematic review of 30 studies concluded that ABSI was superior to BMI and WC in predicting all-cause mortality, but inferior in predicting chronic diseases like CVD [37]. ABSI and CI are the weakest indicators for screening MetS in hemodialysis patients [38]. In our study, the diagnostic ability of ABSI was relatively weak. The diagnostic ability of CI is stronger than that of ABSI, and there is no difference in the diagnostic ability of CI and WHtR in females. ABSI and CI calculations are more complicated than WHtR, but the diagnostic ability is indeed not as good or similar to WHtR. Therefore, ABSI and CI are not recommended for screening Mets in this population.

After adjustment for confounding factors, the TyG index showed the strongest association with MetS in different gender. The associations of all indicators with males (except LAP) were stronger than females, which is consistent with the common knowledge that males have more visceral fat accumulation. Visceral obesity is more common in males and is more harmful to health [5]. Therefore, males should be the key group for primary prevention. In addition, the diagnostic ability of the nomogram models is stronger than that of a single indicator, and the application of multi-indicator joint construction of the model can significantly improve the accuracy of the identification.

The participants in our study were normal-weight adults in rural areas of Xinjiang. Several studies [16–18] have concluded that living in rural areas is a risk factor for MetS relative to living in urban areas. The lifestyles, income levels, and access to health resources of residents living in rural areas and urban areas are quite different. In addition, the prevalence of MetS also differs between rural and urban areas [16–18], so the conclusions of this study are more suitable for extrapolation to rural areas with relatively poor economic status rather than urban areas.

This study evaluates the diagnostic ability of various obesity-related indicators on MetS for normal-weight adults in rural Xinjiang. Questionnaire surveys, physical examinations, and blood biochemical tests were all subject to strict quality control to ensure the quality of the data in this study. This study supplements the evidence for the ability of each indicator to identify MetS among normal-weight populations and provides theoretical support for early screening of MetS in the residents of this area.

There are some limitations in this study. First, this research was a cross-sectional study, we can only report correlations, and there is limited ability to infer causal pathways. Second, we only controlled for confounders such as age, education, occupation, marital status, smoking, and drinking habits. There are still potential confounders that have not been taken into account due to limitations of research capacity. Third, the participants in this study were all rural residents in underdeveloped areas, and the results may not be suitable for extrapolation to urban areas. Further prospective cohort studies with large sample sizes and more detailed data are needed to further evaluate the identification value of each indicator in normal-weight populations.

## Conclusion

LAP and TyG index are effective indicators for identifying MetS among normal-weight adults in rural areas of Xinjiang, which can be widely used in large-scale population screening. Nomogram models including age, CI, LAP, and TyG index can significantly improve diagnostic ability.

## Data Availability

The datasets used during the current study are available from the corresponding author on reasonable request. The Chinese questionnaire copy may be requested from the authors.
